# Optimization of Preanalytical Variables for cfDNA Processing and Detection of ctDNA in Archival Plasma Samples

**DOI:** 10.1155/2021/5585148

**Published:** 2021-07-08

**Authors:** Marijana Nesic, Julie S. Bødker, Simone K. Terp, Karen Dybkær

**Affiliations:** ^1^Department of Hematology, Aalborg University Hospital, Denmark; ^2^Department of Clinical Medicine, Aalborg University, Denmark; ^3^Clinical Cancer Research Centre, Aalborg University Hospital, Denmark

## Abstract

DNA released from cells into the peripheral blood is known as cell-free DNA (cfDNA), representing a promising noninvasive source of biomarkers that could be utilized to manage Diffuse Large B-Cell Lymphoma (DLBCL), among other diseases. The procedure for purification and handling of cfDNA is not yet standardized, and various preanalytical variables may affect the yield and analysis of cfDNA, including the purification kits, blood collection tubes, and centrifugation regime. Therefore, we aimed to investigate the impact of these preanalytical variables on the yield of cfDNA by comparing three different purification kits DNeasy Blood & Tissue Kit (Qiagen), QIAamp Circulating Nucleic Acid Kit (Qiagen), and Quick-cfDNA Serum & Plasma Kit (Zymo Research). Two blood collection tubes (BCTs), EDTA-K2 and Cell-Free DNA (Streck), stored at four different time points before plasma was separated and cfDNA purified, were compared, and for EDTA tubes, two centrifugation regimes at 2000 × *g* and 3000 × *g* were tested. Additionally, we have tested the utility of long-term archival blood samples from DLBCL patients to detect circulating tumor DNA (ctDNA). We observed a higher cfDNA yield using the QIAamp Circulating Nucleic Acid Kit (Qiagen) purification kit, as well as a higher cfDNA yield when blood samples were collected in EDTA BCTs, with a centrifuge regime at 2000 × *g*. Moreover, ctDNA detection was feasible from archival plasma samples with a median storage time of nine years.

## 1. Introduction

Diffuse Large B-Cell Lymphoma (DLBCL) accounts for 30–40% of all newly diagnosed non-Hodgkin lymphoma cases [1]. The first-line therapy for DLBCL is combined immunochemotherapy consisting of rituximab, cyclophosphamide, doxorubicin, vincristine, and prednisone (R-CHOP). Sequencing studies support that DLBCL is a molecularly heterogeneous disease where genetics may have an essential role in patient risk stratification and treatment guidance [2–5]. Tissue biopsies are currently used to diagnose DLBCL patients with the genetic limitation that the biopsied tissue might not represent the whole somatic mutational profile of individual patients due to the existence of subclonal mutations and metastasis [6]. Also, obtaining several biopsies during follow-up is usually unfeasible in clinical practice once the response is achieved due to its invasive nature and diminished or undetectable tumor size [6]. To overcome these obstacles, cell-free DNA (cfDNA) can potentially be used as a malignant DNA source, genuinely representing the whole genetic profile of individual DLBCL patients.

The cfDNA consists mainly of double-stranded DNA fragments with lengths of approximately 180 bp, reflecting the segment of DNA wound around a histone octamer but can also be shorter double-stranded fragments, highly degraded fragments, or partially single-stranded fragments of DNA circulating extracellularly in body fluids [7]. cfDNA is released from normal cells and tumor cells by multiple mechanisms such as apoptosis, necrosis, and active secretion [8, 9], and it is a promising source of biomarkers [8–11]. cfDNA is present in higher amounts in cancer patients than in healthy individuals ranging from 5 to 1500 ng and 1 to 10 ng per ml plasma, respectively [12–14]. cfDNA, which originates from tumor cells, also called circulating tumor DNA (ctDNA), can be isolated together with cfDNA from the patient's blood plasma or serum. ctDNA can be used to identify genetic alterations such as tumor-specific mutations, copy number variations, structural variations, and chromosomal aberrations [15]. The ctDNA amounts correlate with size, localization of tumors, and stage of disease [16]. ctDNA can be used for diagnosis, monitoring of disease progression, prognostic evaluation, and minimal residual disease assessment where tracking the tumor clonotypic immunoglobulin gene rearrangement is highly useful in DLBCL [17]. A recent next-generation sequencing (NGS) study has shown that in DLBCL, cfDNA genotyping can be used with acceptable accuracy to monitor treatment-resistant clones and detect somatic mutation above 20% of variant allele frequency [6].

It has been shown that ctDNA fragments are shorter than normal cfDNA fragments ranging from 90 to 150 bp detected in blood plasma [18].

Detecting ctDNA from blood plasma and serum is challenging as it accounts only for a small fraction (less than 1%) of the total amount of cfDNA, and preanalytical processes including blood collection, purification, and storage are not yet fully standardized [19]. For ctDNA analysis, blood plasma is preferred over serum due to less contamination with genomic DNA (gDNA) by blood cell lysis, which occurs during clotting (17). The major challenge during processing, storage, or transportation of blood samples is the risk of cell lysis resulting in gDNA release, which contaminates the cfDNA. However, this can be prevented by choosing an adequate blood collection tube (BCT) and establishing an optimized approach for blood sample processing [20]. Another essential consideration is the purification method of cfDNA that should provide an optimal yield, and today, the most used purification methods are based on magnetic beads or silica-based membranes.

In this study, two types of commercial BCTs were evaluated for the ability to prevent cfDNA contamination caused by gDNA release as detected by the Agilent 2100 Bioanalyzer (Agilent Technologies). We have also examined three cfDNA purification kits and the usability of archival matched plasma and tumor tissue biopsies assessed by digital droplet Polymerase Chain Reaction (ddPCR). This study is aimed at guiding optimization of preanalytical cfDNA processing variables, ensuring reliable detection of ctDNA.

## 2. Materials and Methods

### 2.1. Clinical Samples

Peripheral blood from each healthy donor (*n* = 8) was collected in EDTA and Streck blood collection tubes in agreement with the Health Ethics Committee of Region North Denmark.

Paired tumor biopsy and matched blood plasma samples were obtained from DLBCL patients (*n* = 15) from the Department of Hematology, Aalborg University Hospital, Denmark. Samples were collected at the time of diagnosis (*n* = 9) or relapse (*n* = 6) and were all part of research protocols RetroSeq (approval jr. N-2014009) and ProSeq (approval jr. N-20160089) approved by the Health Ethics Committee of Region North Denmark. All samples were analyzed by whole-exome sequencing (WES) [21]. Blood samples were collected in EDTA BCTs and processed either the same day (*n* = 6) or within 24 hours (*n* = 9) after the blood was drawn. The median storage time of tissue biopsies and corresponding plasma samples was nine years, ranging from 9 to 14 years for diagnostic samples and 3 to 9 years for relapsed samples.

### 2.2. Optimization of cfDNA Purification

To examine the impact of purification kits on the yield of cfDNA, three DNA purification kits were compared: DNeasy Blood & Tissue Kit (Qiagen), QIAamp Circulating Nucleic Acid Kit (Qiagen), and Quick-cfDNA Serum & Plasma Kit (Zymo Research). cfDNA was purified from blood samples collected from healthy volunteers (*n* = 3). Purification of cfDNA using the QIAamp Circulating Nucleic Acid Kit (Qiagen), Quick-cfDNA Serum & Plasma Kit (Zymo Research), and DNeasy Blood & Tissue Kit (Qiagen) was performed following the manufacturer's instructions. cfDNA was eluted in 50 *μ*l elution buffer. Assessment of cfDNA size and yield was performed by Bioanalyzer and Qubit analysis.

### 2.3. Genomic DNA (gDNA) and Cell-Free DNA (cfDNA) Purification

Purification of gDNA from tumor tissue biopsies was performed using AllPrep DNA/RNA Mini Kit (Qiagen) following the manufacturer's instructions as described previously [21]. Purification of cfDNA from archived blood plasma was achieved by the QIAamp Circulating Nucleic Acid Kit (Qiagen). Samples were purified following the manufacturer's instructions. cfDNA was eluted with 50 *μ*l DNA elution buffer, and concentration was determined immediately after purification using the Qubit dsDNA High-Sensitivity (HS) Assay Kit (Thermo Fisher Scientific) followed by storage at −20°C.

### 2.4. Optimization of cfDNA Processing

**Figure 1:**
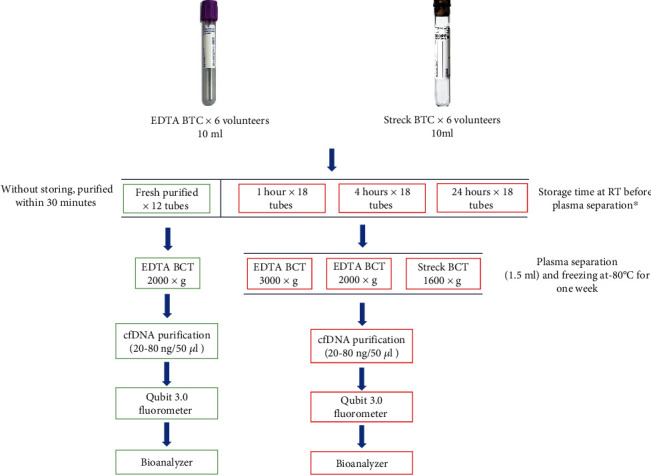
Workflow and experimental design for evaluating cfDNA yields affected by blood collection tubes (BCTs), storage times, and centrifugation regimes. RT: room temperature. Fresh purified samples are framed by green color while samples stored for 1 h, 4 h, and 24 h before plasma separation and freezing are framed with red color. ^∗^The storage at RT refers to EDTA BCTs, which were at 4°C for 24 h before plasma separation. Each step is performed in technical duplicates obtained from the same BCT for corresponding storage time and centrifugation regime.

To investigate the impact of blood collection tubes on cfDNA yield and stability during blood sample storage, each healthy volunteer (*n* = 6) donated 7 × 10 ml blood in K2 EDTA (BD, Franklin Lakes, New Jersey) BCTs and 4 × 10 ml Cell-Free DNA Streck BCT (Streck, Omaha, Nebraska). Blood samples in Streck BCTs were purified within 30 minutes (fresh plasma) from a blood draw or after storing it at room temperature (RT) for one, four, or 24 hours after a blood draw, followed by plasma separation and freezing at −80°C for one week before purification (1 h, 4 h, and 24 h frozen plasma) (Figure 1). For the blood samples in EDTA BCTs, the same time points were used as for Streck BCTs with the difference for samples that were stored for 24 h at 4°C, before plasma separation and freezing at −80°C until purification. Blood samples from each storage time point collected in Streck BCTs were processed as recommended by the manufacturer using a double spin protocol for maximum plasma recovery [22]. Thus, blood samples in Streck BCTs were centrifuged at 1600 × *g* at RT for 10 minutes (Eppendorf centrifuge); plasma was collected by pipetting and centrifuged at 16000 × *g* for 10 minutes. Blood samples collected in EDTA BCTs were centrifuged for 10 minutes at 2000 × *g* or 3000 × *g* at RT for each storage time. After separation of plasma, for both types of tubes, cfDNA was immediately purified from 1.5 ml aliquots of plasma by the QIAamp Circulating Nucleic Acid Kit (Qiagen) following the manufacturer's instruction. The fragmentation size of cfDNA in blood plasma was examined by a Bioanalyzer, and cfDNA concentration was determined by the Qubit dsDNA High-Sensitivity (HS) Assay Kit (Thermo Fisher Scientific).

### 2.5. Droplet Digital PCR (ddPCR)

To investigate the detection of ctDNA in archived DLBCL clinical samples (*n* = 15) by ddPCR, purified plasma samples were eluted in 50 *μ*l elution buffer provided with the QIAamp Circulating Nucleic Acid Kit (Qiagen) and stored at 4°C until ddPCR analysis. Positive control samples containing both wild-type (WT) and mutant (MT) DNA were created for each assay by purifying gDNA from DLBCL cell lines and mixing it with gBlocks purchased from Integrated DNA Technologies (IDT). For each assay, at least three wells with nontemplate controls (NTCs) were used to control environmental contamination, and at least five wells with wild-type controls (WTC) (DLBCL cell lines without the mutation of interest) were used to estimate false-positive rates. The limit of detection was determined for each assay by examining 30-40 wells of cell line gDNA. Reaction volume of 22 *μ*l for ddPCR was prepared by 13 *μ*l master mix (12 *μ*l Supermix for Probes (No dUTP), 1 *μ*l of primer/probe mix for both FAM and HEX) and 9 *μ*l template cfDNA from patient plasma. For NTCs, 9 *μ*l of nuclease-free water was added instead of plasma-purified cfDNA. WTC was generated by adding 9 *μ*l of WT cell line gDNA. The positive controls were generated as ~30% fractional abundance of MT and included in each assay to facilitate thresholding. Wells with total droplet counts of less than 11,000 were considered invalid and were excluded from analysis, ensuring high-quality data. From the PCR mixture, 20 *μ*l was used for droplet generation according to the manufacturer's instructions, and ddPCR was performed using the QX200 ddPCR system (Bio-Rad Laboratories). The QuantaSoft (Bio-Rad Laboratories) software was used for data analysis. For each patient, cfDNA was analyzed in duplicates, and ddPCR results were automatically calculated by the QuantaSoft software based on the mean from estimated target DNA concentrations (copies/*μ*l) in merged wells.

### 2.6. Statistical Analysis

The statistical analysis and the generation of figures were conducted using GraphPad Prism (Version 7, GraphPad Software Inc., La Jolla, CA). Statistical tests performed were a one-way ANOVA test and Wilcoxon signed-rank test.

## 3. Results

To investigate the effects of preanalytical steps on the quantity of cfDNA in plasma samples, purification kits, blood collection tubes, storage time before processing whole blood, and centrifugation regimen were tested as variables for cfDNA yield and quality. Usability of optimized preanalytical steps was applied on archival tumor tissue and plasma samples (*n* = 15) to validate the presence of known somatic mutations in tumor tissue and ctDNA.

### 3.1. Evaluating Three cfDNA Purification Kits for cfDNA Yield

**Figure 2:**
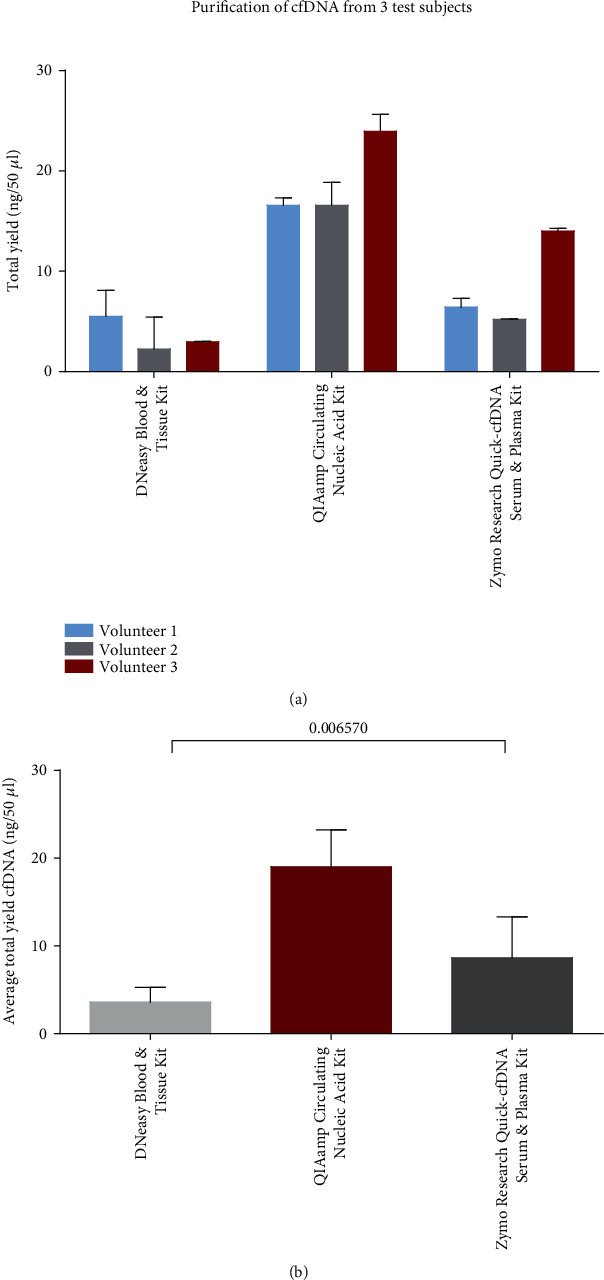
Comparison of cfDNA purification kits. (a) The mean of cfDNA yield of duplicate purification for each volunteer and each kit with a standard deviation. (b) The mean yield of cfDNA from all three volunteers with standard deviation. *P* value obtained by one-way ANOVA test. Bars represent the total yield of cfDNA purified from 1 ml of plasma in technical duplicates.

The yield of cfDNA from blood plasma was assessed using three different DNA purification kits: DNeasy Blood & Tissue Kit (Qiagen), QIAamp Circulating Nucleic Acid Kit (Qiagen), and Quick-cfDNA Serum & Plasma Kit (Zymo Research). cfDNA was purified from blood samples drawn from three healthy volunteers who simultaneously had 3 × 10 ml peripheral blood drawn for each purification kit, and cfDNA was purified from plasma in duplicates. The yield of purified cfDNA was significantly higher from all three volunteers when using the QIAamp Circulating Nucleic Acid Kit compared to the Quick-cfDNA Serum & Plasma Kit and DNeasy Blood & Tissue Kit (Figure 2(a), summarized in Figure 2(b)). The Quick-cfDNA Serum & Plasma Kit also yielded a fair amount of cfDNA, but the DNeasy Blood & Tissue Kit, developed for the purification of higher DNA concentrations, yielded a significantly lower cfDNA amount making downstream analysis difficult. Assessment of the fragmentation size of cfDNA by the Bioanalyzer identified DNA fragments with sizes around 150-180 bp, indicating the enrichment of double-stranded cfDNA and not just purification of contaminating high weight molecular gDNA. Representative microcapillary electropherograms are shown in Figure 1S.

### 3.2. Impact of Blood Collection Tubes and Storage Time on cfDNA Yield

**Figure 3:**
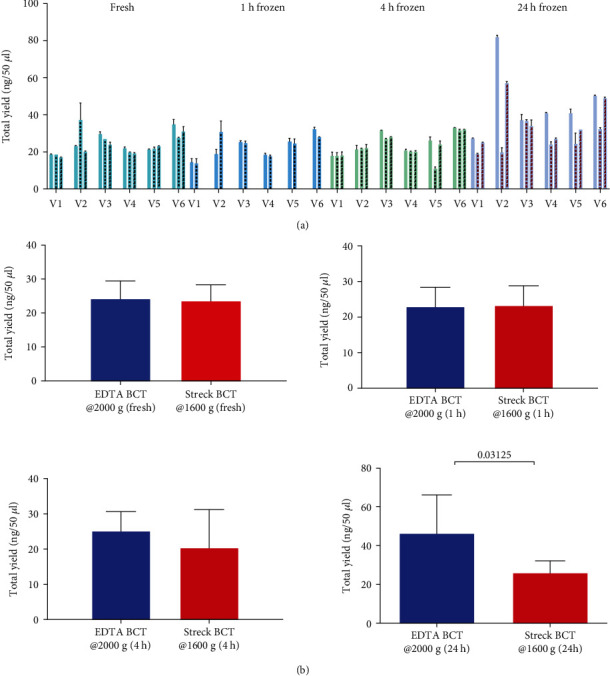
Overview of cfDNA yield at each time point for six volunteers. (a) EDTA BCTs at 2000 × *g* centrifuge regime (first bars without pattern), Streck BCTs at 1600 × *g* centrifuge regime (second bars with pattern), EDTA BCTs at 3000 × *g* (third bars with pattern). Bars represent the mean value of technical duplicates for each time point with a standard deviation. The yield of total cfDNA from each of the volunteers and at each time point is shown in the order of one to six, showing results from both Streck BCTs and EDTA BCTs centrifuged at 2000 × *g*, and 3000 × *g*. (b) The panel shows mean with standard deviation of cfDNA yield for all time points where significant difference in cfDNA yield is between EDTA and Streck BCTs at 24 h time point before plasma separation, freezing, and cfDNA purification (Wilcoxon signed-rank test).

As the yield of cfDNA may be affected by the usage of different BCTs and storage time, we collected blood in EDTA and Streck BCTs from healthy volunteers (*n* = 6) and stored the samples at various conditions before plasma processing (overview in Figure 1). From both types of BCTs, plasma separation was performed less than 30 minutes after the blood draw (fresh), and cfDNA was purified using the QIAamp Circulating Nucleic Acid Kit (Qiagen) following the manufacturer's instruction. To examine the effect of storage time, three different storage time points were tested for both BCTs: one hour (1 h), four hours (4 h), and 24 hours (24 h) from blood draw until plasma was separated and stored at −80°C for one week before cfDNA purification. In both EDTA (centrifuged at 2000 × *g*, RT, 10 min) and Streck BCTs (centrifuged at 1600 × *g*, RT, 10 min), the individual yield of purified cfDNA was determined. For majority of individuals, the total cfDNA yields in EDTA BCTs were slightly increased compared to Streck BCTs at different time points with mean values ranging from 18 to 83 ng/50 *μ*l for EDTA and 11-28 ng/50 *μ*l for Streck, respectively (Figure 3(a)). No significant change in the yield of cfDNA was observed between the two types of BCTs when handling of samples occurred within 30 minutes after blood draw or when stored for one or four hours before plasma separation and freezing (Figure 3(a)). Significance was only observed in cfDNA yield after 24 hours of storage before freezing for EDTA (centrifuged at 2000 × *g*) as compared to Streck BCTs (Figure 3(b)). The Bioanalyzer data show detection of fragments corresponding to the size of cfDNA (Figure 3S).

**Figure 4:**
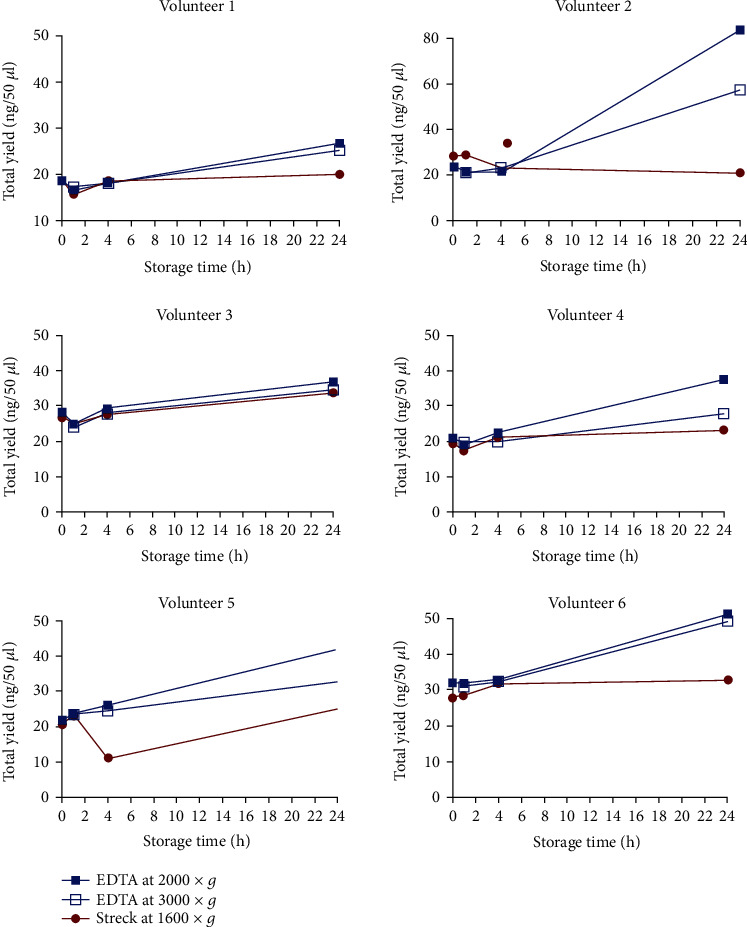
The yield of cfDNA in plasma samples from healthy volunteers drawn in EDTA BCTs and Streck BCTs where the plasma was separated and processed fresh (0 h) and within less than one hour (1 h), after four (4 h), and 24 h storage time. The value of each dot represents the concentration of cfDNA per 50 *μ*l obtained from 1.5 ml plasma and the mean value from technical duplicates.

Less individual variation was observed across time points when blood was drawn in Streck than in EDTA BCTs (Figures 3(a) and 3(b) and Figure 4). The impact of relative centrifugal force on a yield of cfDNA from EDTA BCT was tested using two different relative centrifugation forces of 2000 × *g* and 3000 × *g* for 10 minutes at RT for plasma separation. The cfDNA yield in blood samples from all volunteers was slightly higher in the sample centrifuged at 2000 × *g* for 10 minutes than at 3000 × *g* for 10 minutes at all storage time points (Figure 4).

### 3.3. Detection of ctDNA in Archival Plasma Samples from DLBCL Patients

**Figure 5:**
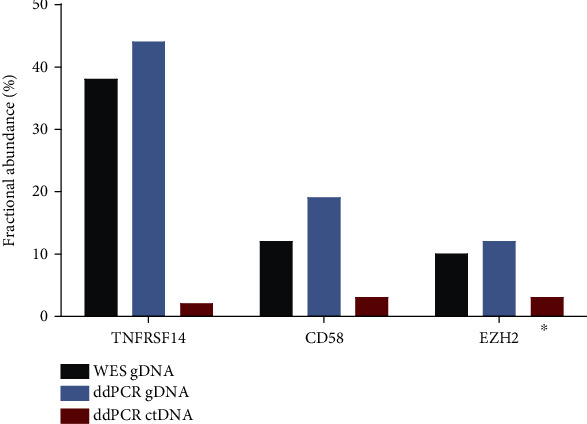
Mutations were identified in ctDNA in three archival clinical samples with known mutations. Fractional abundance of mutations presented in percentage for gDNA assessed by WES and ddPCR and ctDNA assessed by ddPCR. ^∗^The third patient was missing the relapse plasma sample, but we could detect mutation in the diagnostic plasma sample.

To evaluate if cfDNA could be purified from archived material stored in liquid nitrogen (N_2_(l)), 15 plasma samples from patients with DLBCL were purified using the QIAamp Circulating Nucleic Acid Kit (Qiagen). We have used ddPCR to investigate if it is possible to detect previously identified mutations from tumor tissue samples in ctDNA from the same patients. The mutations were identified by WES in the *EZH2*, *CD58*, and *TNFRSF14* genes. All positive tumor tissue samples were positive in the ddPCR analysis of cfDNA when the plasma was extracted from EDTA BCTs and centrifuged by 2000 × *g* (Figure 5 and Figures 3S and 2S).

## 4. Discussion

Standard preanalytical procedures for handling blood samples for cfDNA analysis as well as for the purification of cfDNA have not yet been established for clinical practice. Therefore, this study investigated the impact of selected preanalytical variables on the quantity of cfDNA in plasma samples, including purification kit, storage time before processing whole blood, blood collection tubes, and centrifugation regime. We assessed two parameters for quality control, namely, yield of cfDNA by a Qubit fluorometer and size of cfDNA fragments using the Bioanalyzer. Additionally, ctDNA was detected utilizing ddPCR assays on plasma samples from DLBCL patients with previously identified mutations in archived clinical tumor gDNA, confirming the usability of cfDNA in cancer detection. In addition, for the third patient who harbors a mutation in the *EZH2* gene in the relapse tissue sample, we have detected ctDNA in the diagnostic plasma sample of that patient due to the missing relapse plasma sample.

One of the crucial preanalytical variables that we have investigated is the cfDNA purification kit that can affect the yield of cfDNA considerably. The QIAamp Circulating Nucleic Acid Kit (Qiagen) performed the most efficiently with a significantly increased yield of cfDNA compared to the two other kits investigated, while all three kits performed well in terms of assessed fragmentation size of cfDNA (Figure 1S). The QIAamp Circulating Nucleic Acid Kit also performed consistently in positive and total droplet yields when assessed by ddPCR, which is very important, especially in cancer diagnostics [23]. Notably, the cfDNA yield may differ from study to study using the same purification method due to differences in sample handling or storage temperature [23].

To assess the yield of cfDNA and blood stabilizing capability when using different BCTs, blood samples from six healthy volunteers were stored parallel in two different BCTs for different periods of time and temperatures before plasma was separated. The two types of commercially used BCTs showed similar performance in preserving cfDNA when plasma was freshly processed and when blood was stored for 1 h and 4 h before plasma separation and freezing for one week, followed by cfDNA purification. This observation is in line with the literature where studies recommend processing blood samples drawn in EDTA BCTs immediately or up to two hours from the blood drawn to circumvent the short half-life of cfDNA and contamination by gDNA [23–27]. Nevertheless, cfDNA in the body is cleared through the liver, kidneys, and spleen and by nucleases in the blood, while in BCTs, only nucleases are relevant for potential degradation, which are inactivated in EDTA BCTs [28–31]. In agreement with other studies, we have stored blood samples collected in EDTA BCTs at 4°C for a storage time of 24 hours before plasma separation to prevent cellular gDNA release because it was shown that storage temperature affects the stability of cfDNA in EDTA tubes [20]. However, significantly increased yields of cfDNA were observed in EDTA compared to Streck BCTs after 24 hours of storage, concurring with increased releasing of gDNA from cell lysis of normal hematopoietic cells in the blood sample. For most of the samples, the microcapillary electropherogram data displayed a peak of 150-180 bp corresponding to the size of cfDNA. The centrifugation force can also affect the yield of cfDNA, since strong force may cause white blood cell lysis and thereby contamination with gDNA, whereas too low centrifugation force may lead to a backlog of cellular debris and cells affecting the purification of cfDNA, thus decreasing the yield of cfDNA [23]. For EDTA BCTs, we investigated two centrifugation forces for separation of cfDNA (2000 × *g* and 3000 × *g*) with a consistent slight increase in cfDNA yield at 2000 × *g* becoming more pronounced over time. Therefore, we suggest EDTA BCTs for blood collection if the blood samples can be processed within 24 h and a relative centrifugation force of 2000 × *g* for an optimal yield of cfDNA.

To assess archival plasma samples usability from our biobank, we investigated the presence of ctDNA in archival samples with known rare point mutations in the *CD58*, *TNFRSF14*, and *EZH2* genes using mutation-specific ddPCR assays. The archival blood samples were collected in EDTA BCTs (centrifuged at 2000 × *g* for 10 minutes) and stored in N_2_(I) for a median of nine years. The presence of specific somatic mutations in ctDNA matching tumor gDNA was confirmed, but fractional abundance was decreased compared to gDNA. This was expected due to the dilution of ctDNA in total cfDNA and for our samples, which have been stored in liquid nitrogen for as much as 14 years (average 8.4 years). A recent study has shown that storage of processed plasma up to one year at −80°C does not degrade cfDNA, whereas longer storage results in 30% of degradation per year [32]. However, microcapillary electropherograms of our archival plasma samples displayed the size of cfDNA corresponding to fragments of 100-200 bp, reflecting the release of DNA wound around histone octamer as well as more irregular fragments of single-stranded and partly degraded DNA (Figure 4S).

## 5. Conclusions

In conclusion, our results suggest that QIAamp Circulating Nucleic Acid Kit (Qiagen) as a reliable kit for purification of cfDNA and that blood samples, for which plasma cannot be separated within four hours or stored at 4°C, should be collected in Streck BCTs that can keep cfDNA stable for up to 14 days before processing [26, 27]. Also, we have shown that detecting ctDNA from archival plasma samples with long-term storage is feasible even if they have not been processed fully optimally for ctDNA analysis allowing more uncertainties in especially negative samples on the presence of false-negative samples. Thus, depending on the objective of the ctDNA analysis, minimal residual disease follow-up will depend on thorough and meticulous sample handling procedures, while diagnostic and prognostic assessments relying on ctDNA may, for high abundance mutations, be less sensitive to processing variables.

## Data Availability

The data used to support the findings of this study are included within the supplementary information file.

## Supplementary Materials

Supplementary Materials The supplementary material consists of additional microcapillary electropherograms from the Bioanalyzer and digital droplet PCR (ddPCR) analysis and tables with raw data from ddPCR analysis of archival plasma samples.
