# Supplementary material for: Optimization of Preanalytical Variables for cfDNA Processing and Detection of ctDNA in Archival Plasma Samples
Source: Biomed Res Int. 2021 Jul 8;2021:5585148. doi: 10.1155/2021/5585148 (PMC8285169; doi:10.1155/2021/5585148)
Supplement: Supplementary Materials — The supplementary material consists of additional microcapillary electropherograms from the Bioanalyzer and digital droplet PCR (ddPCR) analysis and tables with raw data from ddPCR analysis of archival plasma samples. [file 5585148.f1.docx]

**SUPPLEMENTARY MATERIAL**

**Optimization of pre-analytical variables for cfDNA processing and detection of ctDNA in archival plasma samples**

**Marijana Nesic ^1,3^, Julie S. Bødker ^1^, Simone K. Terp ^1^, Karen Dybkær ^1,2,3^**

1 Department of Hematology, Aalborg University Hospital

2 Clinical Cancer Research Centre, Aalborg University Hospital

3 Department of Clinical Medicine, Aalborg University

The supplementary material consists of additional micro-capillary electropherograms from Bioanalyzer and digital droplet PCR (ddPCR) analysis and tables with raw data from ddPCR analysis of archival plasma samples.

**SUPPLEMENTARY FIGURES**


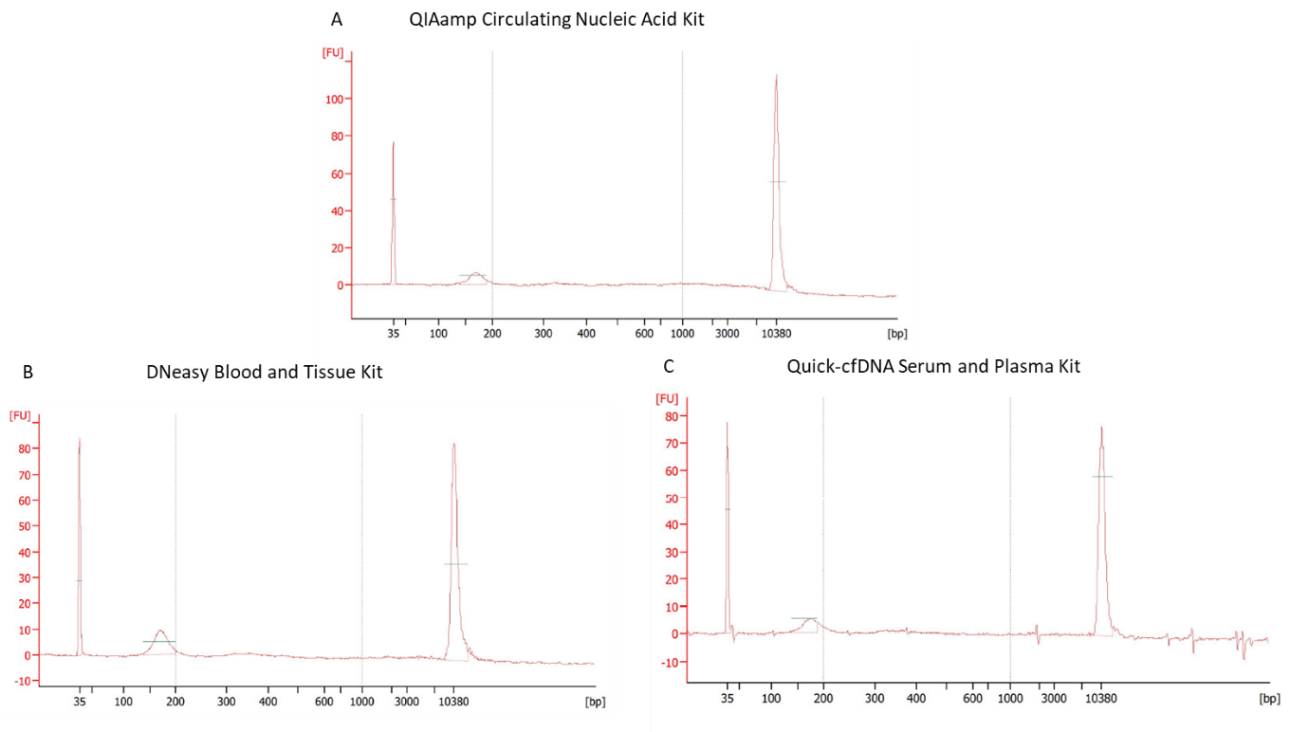


**Figure 1S. Example of** **micro-capillary electropherogram displaying the size of cfDNA** purified with (A) QIAamp Circulating Nucleic Acid Kit, (B) DNeasy Blood & Tissue Kit, and (C) Quick-cfDNA Serum & Plasma Kit. The peak at 35 bp and approximately 10,000 bp correspond to the two size markers used for calculating the fluorescence of cfDNA sizes in the control and unknown samples.


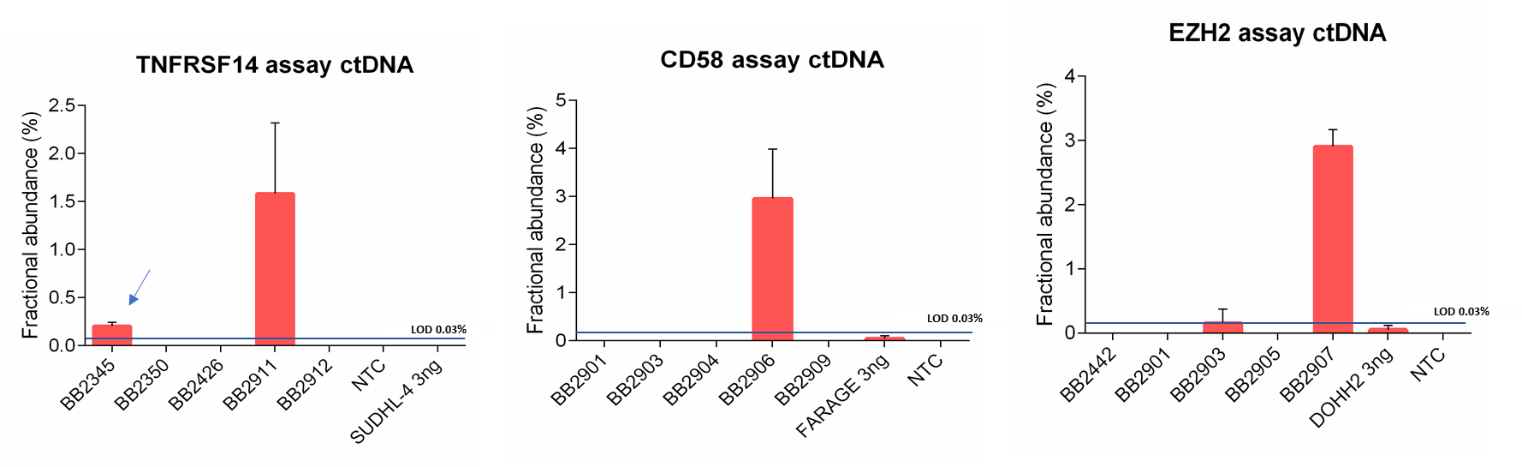
**Figure 2S. Fractional abundance of mutations identified in archival plasma samples by Individual ddPCR assays.** The fractional abundance of mutations in positive samples in percentage is displayed. The sample marked by the arrow was detected negative for the mutation in *TNFRSF14* by WES analysis but positive for a mutation in *TNFRSF14* in ctDNA analysis.


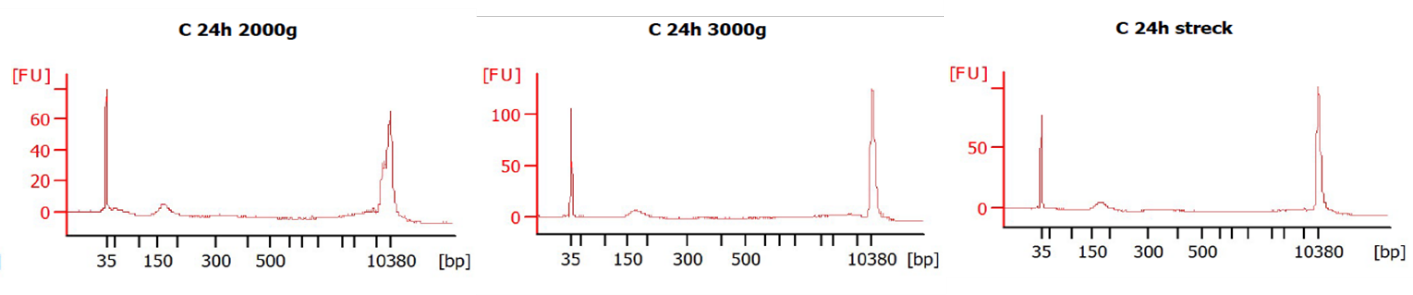


**Figure 3S. Micro-capillary electropherograms from Bioanalyzer.** Examples of fragmentation size of cfDNA from the same volunteer where blood samples collected in EDTA and Streck BCTs had been stored for 24 h storage time before processing, and for EDTA BCT, both centrifugation regimes (at 2000 x g and 3000 x g) are displayed. All plasma samples were, purified with QIAamp Circulating Nucleic Acid Kit (Qiagen).


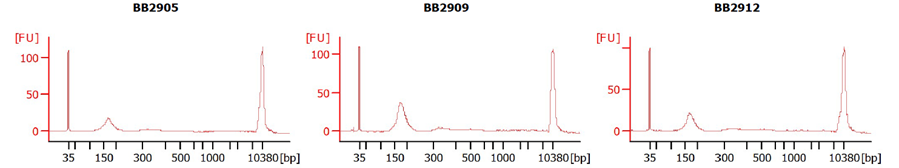


**Figure 4S. Micro-capillary electropherograms from Bioanalyzer.** An example of cfDNA from three different plasma archival clinical samples; Blood was collected in EDTA BCTs and plasma separated at 2000 x g, stored for an average of eight years in liquid nitrogen, purified with QIAamp Circulating Nucleic Acid Kit (Qiagen).

**RAW DATA**

**Raw data for testing the purification kits from plasma samples of n=3 volunteers**

| **Kit used** | **Zymo research Quick -cfDNA serum og plasma kit** | **QIAamp Circulating Nucleic Acid** | **Dneasy blood and tissue** |
| --- | --- | --- | --- |
| Testperson | Total amount in ng | Total amount in ng | Total amount in ng |
| 1a | 7.05 | 17.1 | 7.35 |
| 1b | 5.8 | 16.05 | 3.65 |
| 2a | 5.2 | 14.95 | NA |
| 2b | 5.25 | 18.2 | 4.5 |
| 3a | 14.2 | 25.15 | 3 |
| 3b | 13.8 | 22.8 | 2.9 |
|  |  |  |  |
| a and b (duplicates) | |  |  |

**Raw data for investigation of different time points and blood collection tubes (BCTs) from plasma samples of n=6 volunteers**

|  | Average amount of duplicates (ng) | | | |
| --- | --- | --- | --- | --- |
| Volunteers (V1-6) | 0h - fresh plasma | 1h - frozen plasma | 4h - frozen plasma | 24h - frozen plasma |
| EDTA @2000g-V1 | 18.65 | 16.55 | 18.3 | 26.8 |
| EDTA @3000g-V1 | none | 17.15 | 18.05 | 25.2 |
| Streck @1600g-V1 | 18.6 | 15.75 | 18.7 | 20.05 |
| EDTA @2000g-V2 | 23.5 | 21.05 | 21.35 | 83.6 |
| EDTA @3000g-V2 | none | 20.75 | 22.85 | 57.25 |
| Streck @1600g-V2 | 28.2 | 28.65 | 22.8 | 20.85 |
| EDTA @2000g-V3 | 28.3 | 25.15 | 29.35 | 37.1 |
| EDTA @3000g-V3 | none | 24.05 | 28.05 | 34.75 |
| Streck @1600g-V3 | 27 | 25.15 | 27.55 | 33.9 |
| EDTA @2000g-V4 | 21.1 | 19.1 | 22.45 | 37.55 |
| EDTA @3000g-V4 | none | 19.65 | 20.1 | 27.9 |
| Streck @1600g-V4 | 19.35 | 17.5 | 21.2 | 23.3 |
| EDTA @2000g-V5 | 21.6 | 23.65 | 25.9 | 41.5 |
| EDTA @3000g-V5 | none | 23.3 | 24.3 | 32.45 |
| Streck @1600g-V5 | 20.55 | 23.45 | 11 | 24.6 |
| EDTA @2000g-V6 | 32.3 | 32.05 | 33.15 | 51.5 |
| EDTA @3000g-V6 | none | 31.05 | 32.5 | 49.45 |
| Streck @1600g-V6 | 27.95 | 28.8 | 31.75 | 33.05 |

Raw data from ddPCR tests for clinical samples (3 assays)

| Assay 1 (Gene CD58) | |  |  |  |
| --- | --- | --- | --- | --- |
| Sample | Conc(copies/µL) | Copies/20µLWell | Accepted Droplets | FA % |
| BB2901 | 0.00 | 0.00 | 16410 |  |
| BB2901 | 0.00 | 0.00 | 15321 |  |
| BB2903 | 0.00 | 0.00 | 15866 |  |
| BB2903 | 0.00 | 0.00 | 14273 |  |
| BB2904 | 0.00 | 0.00 | 13750 |  |
| BB2904 | 0.00 | 0.00 | 15724 |  |
| BB2906 | 1.05 | 21.05 | 16775 | 2.22 |
| BB2906 | 1.80 | 35.92 | 13765 | 3.68 |
| BB2909 | 0.00 | 0.00 | 16462 |  |
| BB2909 | 0.00 | 0.00 | 15236 |  |
| FARAGE (WT) | 0.00 | 0.00 | 15552 |  |
| FARAGE (WT) | 0.00 | 0.00 | 15680 |  |
| FARAGE (WT) | 0.00 | 0.00 | 14746 |  |
| FARAGE (WT) | 0.08 | 1.53 | 15415 | 0.14 |
| FARAGE (WT) | 0.00 | 0.00 | 16303 |  |
| Posistive control | 26.66 | 533.16 | 15488 | 51.04 |
| NTC | 0.00 | 0.00 | 15237 |  |
| NTC | 0.00 | 0.00 | 16697 |  |
| NTC | 0.00 | 0.00 | 14563 |  |

| Assay 2 (Gene TNFRSF14) | |  |  |  |
| --- | --- | --- | --- | --- |
| Sample | Conc(copies/µL) | Copies/20µLWell | Accepted Droplets | FA% |
| BB2345 | 0.10 | 2.08 | 11334 | 0.23 |
| BB2345 | 0.07 | 1.45 | 16215 | 0.17 |
| BB2350 | 0.00 | 0.00 | 12312 |  |
| BB2426 | 0.00 | 0.00 | 12696 |  |
| BB2426 | 0.00 | 0.00 | 15046 |  |
| BB2911 | 0.10 | 1.93 | 12181 | 1.06 |
| BB2911 | 0.16 | 3.21 | 14646 | 2.10 |
| BB2912 | 0.00 | 0.00 | 12495 |  |
| BB2912 | 0.00 | 0.00 | 14152 |  |
| BB2912 | 0.00 | 0.00 | 15470 |  |
| SUDHL-4 (WT) | 0.00 | 0.00 | 14200 |  |
| SUDHL-4 (WT) | 0.00 | 0.00 | 14913 |  |
| SUDHL-4 (WT) | 0.00 | 0.00 | 14704 |  |
| SUDHL-4 (WT) | 0.00 | 0.00 | 16088 |  |
| SUDHL-4 (WT) | 0.00 | 0.00 | 15916 |  |
| Posistive control | 5.63 | 112.62 | 15916 | 32.56 |
| NTC | 0.00 | 0.00 | 14526 |  |
| NTC | 0.00 | 0.00 | 15403 |  |
| NTC | 0.00 | 0.00 | 15445 |  |

| Assay 3 (Gene EZH2) | |  |  |  |
| --- | --- | --- | --- | --- |
| Sample | Conc(copies/µL) | Copies/20µLWell | Accepted Droplets | FA% |
| BB2442 | 0.00 | 0.00 | 13760 |  |
| BB2442 | 0.00 | 0.00 | 10939 |  |
| BB2901 | 0.00 | 0.00 | 15238 |  |
| BB2901 | 0.00 | 0.00 | 16572 |  |
| BB2903 | 0.08 | 1.53 | 15348 | 0.31 |
| BB2903 | 0.00 | 0.00 | 13929 |  |
| BB2905 | 0.00 | 0.00 | 14774 |  |
| BB2905 | 0.00 | 0.00 | 14552 |  |
| BB2907 | 0.46 | 9.19 | 12802 | 2.71 |
| BB2907 | 0.53 | 10.67 | 13237 | 3.09 |
| DOHH2 (WT) | 0.00 | 0.00 | 15165 |  |
| DOHH2 (WT) | 0.00 | 0.00 | 13624 |  |
| DOHH2 (WT) | 0.00 | 0.00 | 15007 |  |
| DOHH2 (WT) | 0.00 | 0.00 | 13007 |  |
| DOHH2 (WT) | 0.00 | 0.00 | 12878 |  |
| Posistive control | 12.25 | 244.97 | 13807 | 20.18 |
| NTC | 0.00 | 0.00 | 14363 |  |
| NTC | 0.00 | 0.00 | 14529 |  |
| NTC | 0.00 | 0.00 | 15446 |  |
|  |  |  |  |  |
